# Integrated machine learning-driven disulfidptosis profiling: CYFIP1 and EMILIN1 as therapeutic nodes in neuroblastoma

**DOI:** 10.1007/s00432-024-05630-8

**Published:** 2024-03-01

**Authors:** Zhang Mengzhen, Hou Xinwei, Tan Zeheng, Li Nan, Yang Yang, Yang Huirong, Fan Kaisi, Ding Xiaoting, Yang Liucheng, Wu Kai

**Affiliations:** Department of Pediatric Surgery, Zhujiang Hospital, Southern Medical University, Guangzhou, 510282 Guangdong China

**Keywords:** Disulfidptosis, Neuroblastoma, Pediatric tumor, Machine learning, Molecular subtypes

## Abstract

**Background:**

Neuroblastoma (NB), a prevalent pediatric solid tumor, presents formidable challenges due to its high malignancy and intricate pathogenesis. The role of disulfidptosis, a novel form of programmed cell death, remains poorly understood in the context of NB.

**Methods:**

Gaussian mixture model (GMM)-identified disulfidptosis-related molecular subtypes in NB, differential gene analysis, survival analysis, and gene set variation analysis were conducted subsequently. Weighted gene co-expression network analysis (WGCNA) selected modular genes most relevant to the disulfidptosis core pathways. Integration of machine learning approaches revealed the combination of the Least absolute shrinkage and selection operator (LASSO) and Random Survival Forest (RSF) provided optimal dimensionality reduction of the modular genes. The resulting model was validated, and a nomogram assessed disulfidptosis characteristics in NB. Core genes were filtered and subjected to tumor phenotype and disulfidptosis-related experiments.

**Results:**

GMM clustering revealed three distinct subtypes with diverse prognoses, showing significant variations in glucose metabolism, cytoskeletal structure, and tumor-related pathways. WGCNA highlighted the red module of genes highly correlated with disulfide isomerase activity, cytoskeleton formation, and glucose metabolism. The LASSO and RSF combination yielded the most accurate and stable prognostic model, with a significantly worse prognosis for high-scoring patients. Cytological experiments targeting core genes (CYFIP1, EMILIN1) revealed decreased cell proliferation, migration, invasion abilities, and evident cytoskeletal deformation upon core gene knockdown.

**Conclusions:**

This study showcases the utility of disulfidptosis-related gene scores for predicting prognosis and molecular subtypes of NB. The identified core genes, CYFIP1 and EMILIN1, hold promise as potential therapeutic targets and diagnostic markers for NB.

**Supplementary Information:**

The online version contains supplementary material available at 10.1007/s00432-024-05630-8.

## Introduction

Neuroblastoma (NB), the most common extracranial solid tumor in children, accounts for approximately 8% of all pediatric tumors and 13% of pediatric tumor-related deaths (Maris et al. 2007; Ward et al. 2014). NB is a malignant tumor of the sympathetic nervous system, originating in the developing sympathetic nervous system and commonly found in bilateral adrenal and sympathetic ganglia (Marshall et al. 2014). There are various staging systems for NB tumors based on size and metastasis, such as The International Neuroblastoma Staging System (INSS) (Brodeur et al. 1988; Castel et al. 1999) and the Children’s Oncology Group (COG) staging system (Bagatell et al. 2023; Pinto et al. 2019). NB is categorized as low, medium, or high risk, with favorable outcomes for low and medium risk, often curable through surgery or experiencing spontaneous regression. However, high-risk patients face poor prognosis, with a less than 50% 5-year survival rate (Pinto et al. 2015), and approximately 60% of high-risk patients do not respond to initial treatments, experiencing recurrence or metastasis within 2 years (London et al. 2011; Whittle et al. 2017). Therefore, it is imperative to delve deeper into the pathogenesis of NB and explore novel therapeutic avenues.

Cancer is characterized by dysregulated cell death, leading to rapid tumor growth and metastasis. As research into tumor cell death deepens, various novel death modes have been discovered, providing new therapeutic targets for tumors. Recently, Liu et al. (2023a, b) discovered a novel form of regulated cell death (RCD) known as disulfidptosis, which occurs when cells are deprived of glucose, upregulation of SLC7A11, and subsequent high uptake of cystine, resulting in the depletion of cytoplasmic NADPH and accumulation of disulfides, ultimately triggers the formation of abnormal disulfide bonds in the actin cytoskeleton proteins and collapse of the actin filament (F-actin) network, leading to cell death. This new mode of cell death has been found to affect the occurrence and progression of various tumors, such as hepatocellular carcinoma and thyroid cancer (Feng et al. 2023; Wang et al. 2023), but its role in NB remains rarely reported.

High-throughput sequencing technology has facilitated the identification of biological characteristics in various tumors (Reuter et al. 2015). However, discerning pathogenic and protective genes from massive data is not an easy task. Bioinformatics, an amalgamation of biology with mathematics and computers, facilitates efficient data analysis and is progressively applied in medicine (Jiménez-Santos et al. 2022). The integration of genomics combined with deep machine learning plays an increasingly important role in disease diagnosis, prognosis assessment, and mechanism exploration (Greener et al. 2022). Numerous prognostic models for NB, utilizing diverse gene expression profiles, have been constructed to assess the disease risk and prognosis, contributing to the formulation of personalized diagnosis and treatment strategies. However, despite being the most prevalent pediatric malignancy, limited research has been conducted on the prognostic significance of genes associated with disulfidptosis in NB, and experimental exploration is yet to be undertaken (Zhu et al. 2023).

This study aims to analyze the expression of disulfidptosis-related genes in NB, explore the molecular subtypes, construct a prognostic model, identify potential therapeutic targets, guide personalized diagnosis and treatment for NB patients, and provide experimental evidence for further research.

## Materials and methods

### Collection of NB datasets

Clinical data and RNA sequencing data regarding NB patients were acquired from the Gene Expression Omnibus (GEO; https://www.ncbi.nlm.nih.gov/geo/) database, with 498 samples (GSE49710) included and set as the TRAIN dataset. From the Therapeutically Applicable Research to Generate Effective Treatments (TARGET; https://ocg.cancer.gov/programs/target) database, 247 samples were acquired and set as the TEST dataset, 2 patients without survival information were excluded. The two datasets were combined using the COMBAT function of the "SVA" R package to remove the batch effect and obtain the ALL dataset with 745 samples.

### Gaussian mixture model (GMM) clustering for disulfidptosis-related genes in NB

According to literature reports, 10 disulfidptosis genes including GYS1, OXSM, LRPPRC, NDUFA11, NUBPL, RPN1, SLC3A2, SLC7A11, and NCKAP1 were selected for analysis (Liu et al. 2023a, b). Based on the expression level of these 10 genes, 498 NB cases from GEO were classified using the GMM clustering analysis. Principal component analysis was employed to determine the differentiation of each subtype. The R package “mclust” was used to determine the number of clusters. The Kaplan–Meier method was used to estimate the difference in overall survival between different clusters. Log-rank test was performed to assess survival differences. The DEGs between three clusters were analyzed using the R package “limma”.

### Gene set variation analysis (GSVA) enrichment analysis of cellular functions and pathways

The R package “GSVA” was used to identify different Gene ontology (GO) terms and Kyoto Encyclopedia of Genes and Genome (KEGG) pathways to analyze the differences in biological function among the different disulfidptosis clusters. The gene sets of “c2.cp.kegg.v2023.1.Hs.symbols.gmt” and “c5.go.v2023.1.Hs.symbols.gmt” were downloaded from the MSigDB database to run GSVA enrichment analysis. The expression of disulfidptosis-related pathways and some classical tumor-associated signaling pathways was presented as heatmaps.

### Weighted gene co-expression network analysis (WGCNA)

WGCNA analysis was carried out using the "WGCNA" software package. The differential gene matrix between cluster 1 and cluster 3 was used for WGCNA analysis to obtain modules related to glucose metabolism, cytoskeleton, and disulfide isomerase. Automated networks were used to create co-expression networks, which were detected using hierarchical clustering and dynamic tree-cutting functions in this module. Module membership and gene significance were used to connect the modules to the GSVA enrichment results, and the modules most relevant to disulfidptosis-related pathways such as glucose metabolism, disulfide isoenzymes, and cytoskeletal changes were filtered for subsequent analyses.

### Construction of NB prognostic models with multiple machine learning

Various machine learning methods including CoxBoost, Gradient Boosting Machine (GBM), plsRcox, Random Survival Forest (RSF), stepCox, superPC, and survival Support Vector Machine (survival-SVM) were, respectively, combined with the Least absolute shrinkage and selection operator (LASSO) by employing “CoxBoost”, “gbm”, “plsRcox”, “randomForestSRC”, “BART”, “superpc”, “survivalsvm”, and “glmnet” R packages, followed by the use of the “compareC” R package to compare the C-index of the prognostic models obtained from different combinations and select the model with the highest prediction accuracy. The TRAIN dataset was used for training, and the TEST and ALL datasets were employed for external validation. After comparing different machine learning combination methods, the model obtained by combining LASSO and RSF with the best prediction accuracy was finally chosen, which contains 44 genes.

The final prognostic model was constructed using the "randomForestSRC" and "glmnet" R packages and can be succinctly summarized by the following equation:

$${\text{risk}}\;{\text{score}} = \mathop \sum \limits_1^n {\text{Coefficient}}\left( {{\text{gene}}_n } \right) \times {\text{Expression}}\left( {{\text{gene}}_n } \right).$$

### Validation of NB prognostic models

Patients were divided into high-risk and low-risk groups based on the optimal cut-off value calculated by the “Survminer” R package. Survival analyses were performed to assess the prognosis of patients in different groups. The ROC curve was plotted using the “survivalROC” R package, the area under the curve (AUC) was calculated, and the predictive power of the feature was assessed.

### Clinical specimens

Collecting tissue slices from the pathology department of Zhujiang Hospital (8 cases, from May 2022 to August 2023) for immunohistochemical identification. The research protocol has obtained approval from the Ethics Committee of Zhujiang Hospital, Southern Medical University. Specimen collection has been conducted with informed consent from the guardians of the pediatric patients.

### RT-qPCR

Total RNA was isolated with TRIzol reagent, and cDNA was reversed using Evo-M-MLV RT kit and gDNA clean qPCR II Reverse Transcription Kit (Accurate Biology, China). The SYBR Green Premix Pro Taq HS qPCR kit (Accurate Biology, China) was used for qPCR assays using the human vinculin gene as an internal reference. The qPCR amplification procedure was performed in the following three-step manner: pre-denaturation at 95 °C for 5 min once, followed by 40 cycles (denaturation at 95 °C for 10 s, annealing at 50–60 °C for 30 s), and extension data collection at 72 °C for 30 s. Relative expression levels of target genes were detected by the Bio-RadCFX96 Touch (Bio-Rad, USA) and analyzed using ∆∆CT method. The sequences of all primers (RuiBioTech, China) are listed in Table 1.

**Table 1:** The sequences of primers for RT-qPCR

Gene name	Primer sequence (5′–3′)
CYFIP1	Forward: GTTCCTGTACGACGAAATTGAGG
	Reverse: GTGGCTCCCTGATTCTTGC
EMILIN1	Forward: GGGCCGACTAGAGCAGTTG
	Reverse: CTGAGGATCTCGCTGACTTGA
Vinculin	Forward: CTCGTCCGGGTTGGAAAAGAG
	Reverse: AGTAAGGGTCTGACTGAAGCAT

### Western blot

Proteins were extracted with RIPA buffer (Servicebio, China) and quantified by BCA analysis. Equal quantities of proteins were separated by 8% SDS-PDS gel electrophoresis and transferred to a PVDF membrane (Immobilon, Germany). After blocking with 5% skim milk powder and washing with TBST, the membranes were incubated with anti-CYFIP1 (1:1000, bs-14139R, Bioss, China), anti-vinculin (1:2000, AB-2936321, Abmart, China), and anti-EMILIN1 (1:1000, sc-365737, SantaCruz, USA) antibodies overnight at 4 °C; then the prepared membranes were incubated with second antibodies for 1 h at room temperature. Finally, Immobilon ECL Ultra Western HRP substrate was added to detect the protein blot and the relevant data were analyzed using ImageJ software.

### Immunohistochemistry staining

Paraffin NB tissue slices were dewaxed and hydrated, then incubated in endogenous peroxidase enzymes blocking buffer for 10 min at room temperature, then blocked with Normal Goat Serum for 10 min, and dried. The slices were incubated overnight at 4 °C with CYFIP1 (1:1000, bs-14139R, Bioss, China) and EMILIN1 (1:1000, sc-365737, SantaCruz, USA) antibodies, washed, and then incubated with secondary antibody working solution for 10 min at room temperature and washed thoroughly, then Streptavidin-HRP was added and incubated for 10 min at room temperature. Staining was done with DAB chromogenic working solution. After sealing the sections, photographs were taken with a 3D HISTECH bright-field scanner. All reagents used in this experiment were obtained from SP Rabbit and Mouse HRP Kit (CWBIO, CW2069S, China).

### Cell culture and cell transfection

Human neuroblastoma cells SH-SY5Y(RRID:CVCL_0019) and SK-N-AS(RRID:CVCL_1700) were purchased from HyCyte (Soochow, China) and cells were cultured according to the instructions. Cells were knocked down using si-CYFIP1, si-EMILIN1, and negative control provided by TSINGKE(Beijing, China). The transfection was performed in six-well plates using TSnanofect V2 (TSINGKE, China). siRNA sequences are listed in Table 2.

**Table 2:** siRNA sequences for transfection

Gene name	Sequence (5′–3′)
CYFIP1	Positive-sense strand: CACGUGAUGGAAGUGUAUUTT
	Antisense strand: AAUACACUUCCAUCACGUGTT
EMILIN1	Positive-sense strand: GGCUAUUAUGAUCCAGAGATT
	Antisense strand: UCUCUGGAUCAUAAUAGCCTT

### Colony formation assays

The transfected cells were evenly seeded into a 6-well plate at a density of 1000 cells per well. The cells were then cultured for 14 days, with medium change and cell observation every 2 days. After that, the cells were washed once with PBS, fixed with 4% paraformaldehyde for 15 min, and stained with 1% crystal violet dye to observe colony formation.

### Wound-healing assay

Cells were cultured in six-well plates until evenly spread, and the wells were delineated with a 10 μ lance tip, followed by starvation culture in serum-free medium. Photographs were taken at 0, 24 h, and 48 h with a Zeiss Axio Scope A1 microscope and subsequently analyzed using ImageJ software.

### Transwell assay

Transfected NB cells (2 × 10^4^/ml) were inoculated into Transwell chambers (FALCON, USA) containing 250 μ serum-free medium, and 500 μ serum-containing medium was added to the bottom of the chambers. After 48 h, the chambers were fixed with 4% paraformaldehyde and stained with crystal violet. Five randomly selected fields of view were photographed using Zeiss Axio Scope A1 microscope and cell counting was performed using Image J.

### Actin staining and fluorescence analysis

Transfected cells were seeded onto cell culture slides to achieve a confluency of 50%. The cells were washed and fixed with 4% paraformaldehyde for 10 min. After fixation, the cells were washed three times with PBS and permeabilized with a 0.5% Triton X-100 solution (Solarbio, China) for 5 min. A 200 µL working solution of TRITC-labeled phalloidin (Yeasen, China) was added to cover the cells on the slides, and they were incubated in the dark at room temperature for 30 min. For nuclear counterstaining, the cells were briefly stained with 200 µL of DAPI solution (concentration: 100 nM) for approximately 30 s. The slides were then washed with PBS and coverslips were applied. Finally, fluorescence imaging was performed using the Nikon ECLIPSE Ti2 inverted microscope with TRITC excitation/emission filters (*Ex*/*Em* = 545/570 nm) and DAPI excitation/emission filters (*Ex*/*Em* = 364/454 nm).

### Statistics analysis

All bioinformatic data and images were processed and plotted using RStudio in R software (version 4.2.1, New Zealand). GraphPad (version 9.4.1, USA) and ImageJ (version 1.53e, USA) were used to process the experimental data and images. All statistical p-values were two sided, and *p* < 0.05 was considered a statistically significant difference.

## Results

### Exploring the function of disulfidptosis genes in NB: prognostic implications and Gaussian clustering analysis

**Fig. 1:**
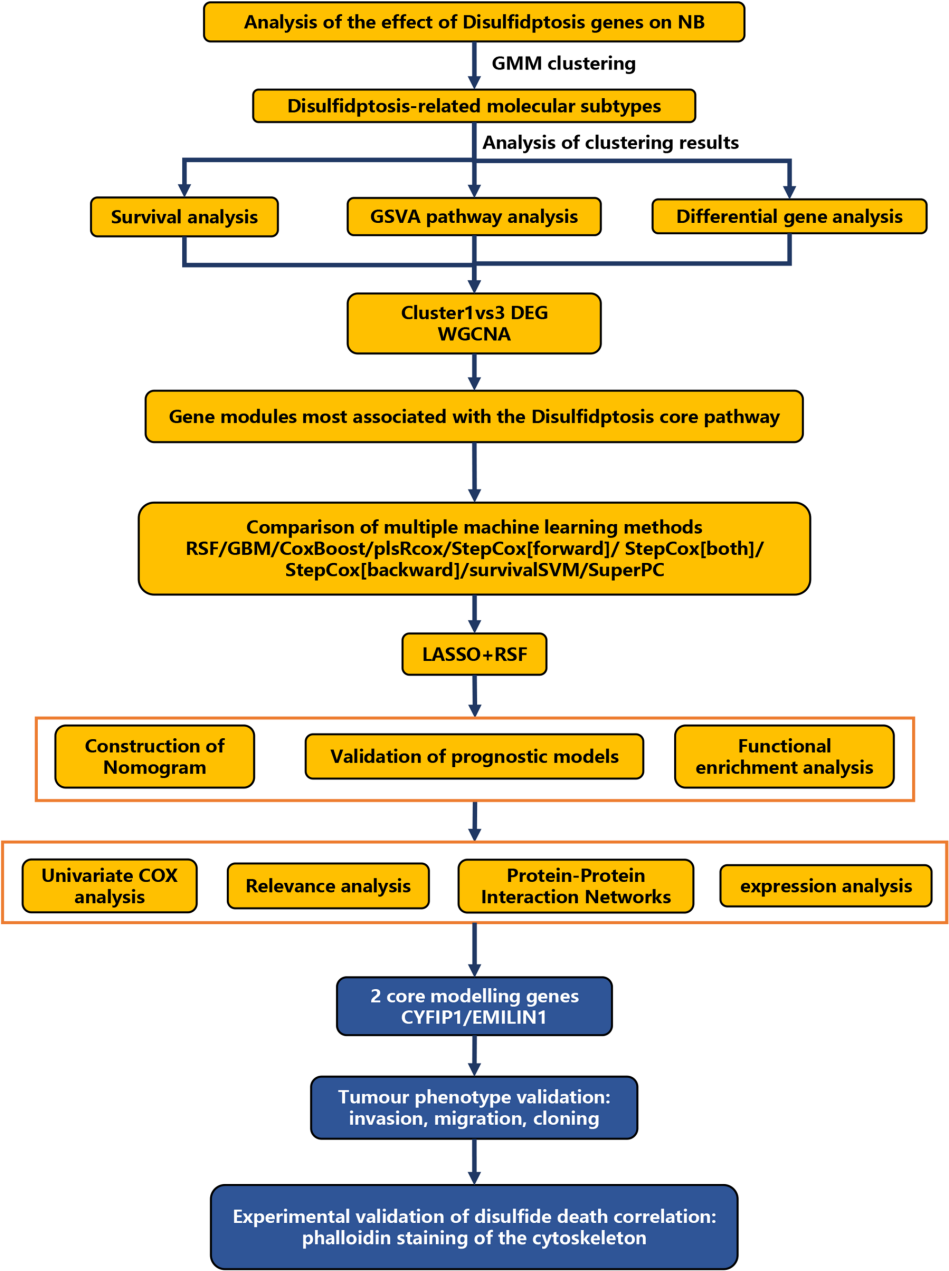
Research overview. Flowchart of this study

**Fig. 2:**
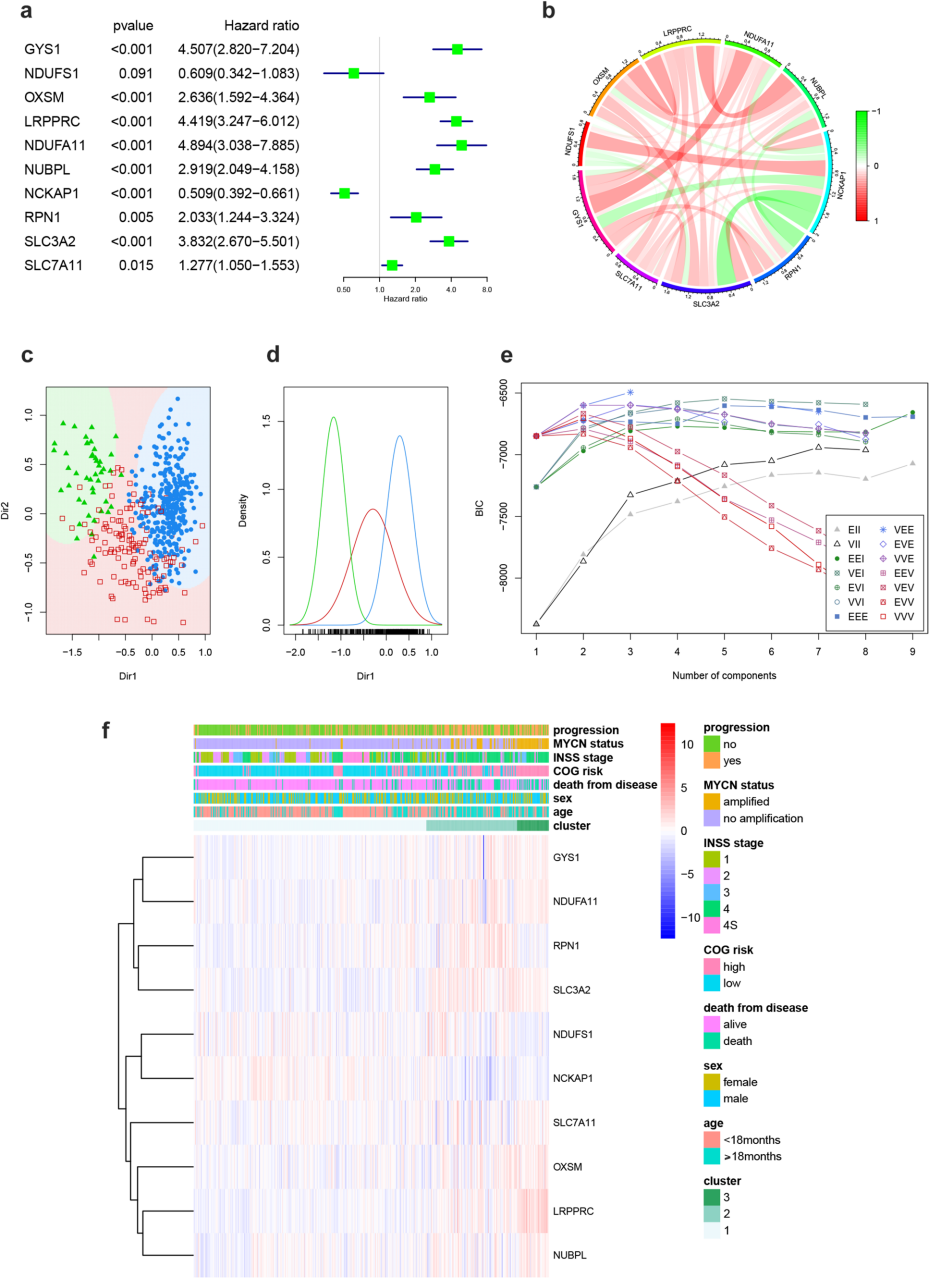
Disulfidptosis genes in neuroblastoma and GMM clustering based on the expression of these genes to obtain molecular subtypes. **a** Univariate Cox analysis of disulfidptosis in the GSE49710 dataset. **b** Circle plot of co-expression relationships of disulfidptosis genes in GSE49710 dataset. **c** Sample distributions of 3 subtypes in Gaussian clustering. **d** Density distribution of 3 subtypes in Gaussian clustering. **e** BIC curves for different subtype numbers. **f** Heatmap comparing clinical characteristics among 3 disulfidptosis molecular subtypes

The flowchart of this study is shown in Fig. 1. To investigate the expression patterns and prognostic impact of 10 disulfidptosis genes in NB, a univariate Cox analysis was performed in conjunction with the GSE49710 dataset (Fig. 2a). The results showed that 8 genes (including GYS1, OXSM, LRPPRC, NDUFA11, NUBPL, RPN1, SLC3A2, SLC7A11) were adverse prognostic factors, while 1 gene (NCKAP1) was a favorable factor. A circle plot (Fig. 2b) and correlation heatmaps (Sup. 1a–b) were generated to visualize the interactions among these genes in the GSE49710 dataset. Based on the expression levels of 10 disulfidptosis genes, GMM clustering analysis (Kageyama et al. 2021; Zhao et al. 2019) was performed on GSE49710, and the optimal clustering result yielded three subtypes (Fig. 2c–e, Sup. 1c). A heatmap (Fig. 2f) comparing the clinical traits between the 3 clusters showed that the proportion of patients with mostly high-risk progression status, N-MYC gene amplification, and poor prognosis was significantly higher in the cluster 3 group.

### Cluster-wise differential gene analysis, survival analysis, and GSVA pathway enrichment

**Fig. 3:**
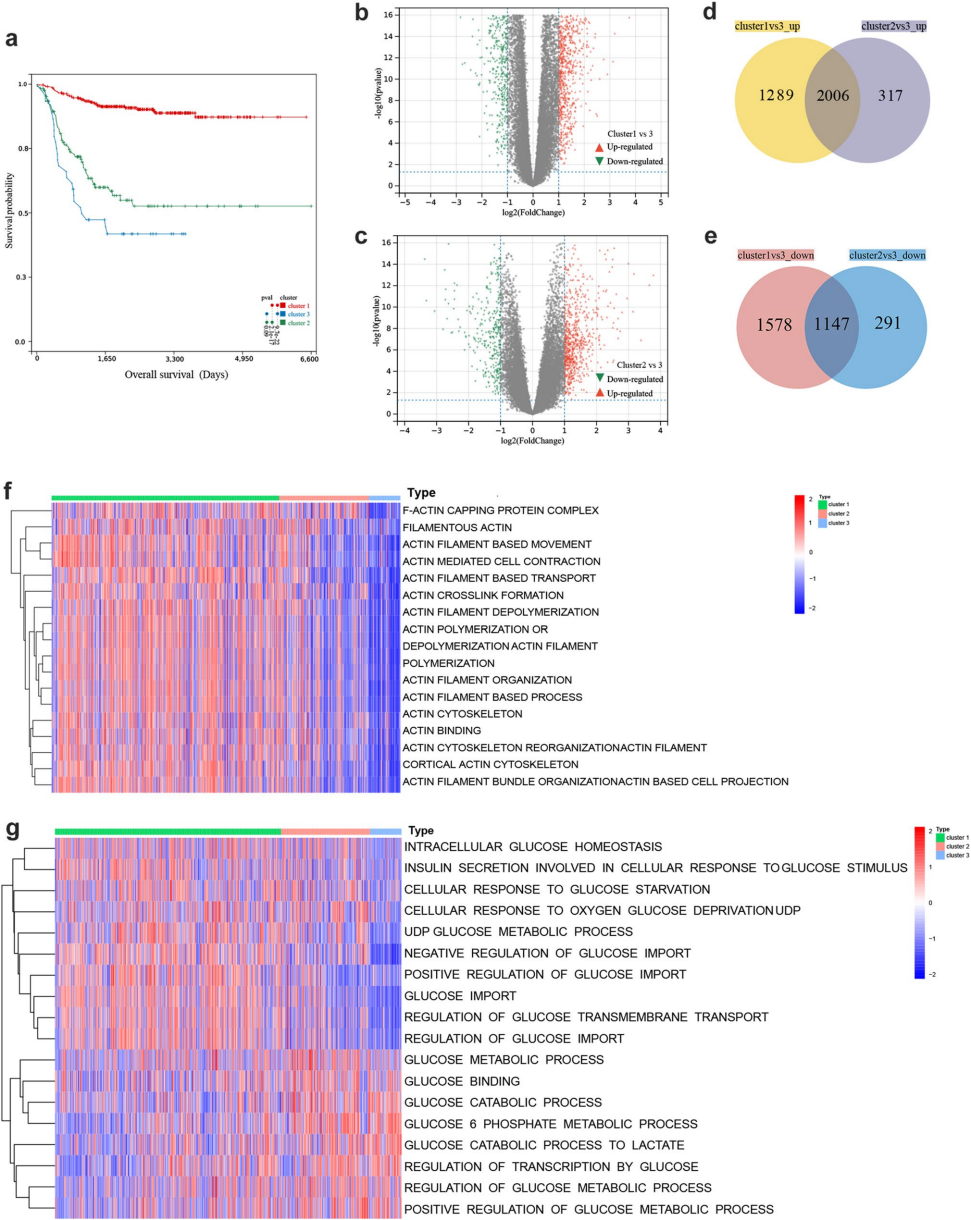
Differential gene analysis and GSVA pathway analysis between 3 disulfidptosis molecular subtypes. **a** Survival analysis among the 3 disulfidptosis subtypes. **b** Volcano plot of differential genes between cluster 1 and cluster 3. **c** Volcano plot of differential genes between cluster 2 and cluster 3. **d**–**e** Venn diagrams showed 2006 genes upregulated and 1147 genes downregulated in all 3 clusters. **f** Heatmap of cytoskeletal actin-related pathways expression in 3 molecular subtype samples. **g** Heatmap of glucose metabolism-related pathways expression in 3 molecular subtype samples

Survival analysis showed significant prognostic differences among the three disulfidptosis subtypes (*p* = 9.7e−22), with cluster 1 having a considerable survival advantage and cluster 3 having the worst prognosis (Fig. 3a), and there was a significant difference in gene expression among the three clusters (Fig. 3b–c, Sup. 2a–b). The Venn diagrams showed that there were 2006 genes upregulated and 1147 genes downregulated in all 3 clusters (Fig. 3d–e). Using the GSVA algorithm, we heatmapped the classical tumor KEGG pathways (Sup. 2c), cytoskeletal and glucose metabolism-related GO pathways (Fig. 3f–g) among the three clusters. Significant differences in pathway expression were observed among the clusters. Cluster 3 exhibited significant downregulation in cytoskeletal proteins (ACTIN-related proteins), glucose import and glucose metabolic pathways. Additionally, there was a trend of downregulation in response to glucose starvation, while showing a positive regulation in glucose catabolic pathways.

### WGCNA unveils modules linked to key pathways in disulfidptosis

**Fig. 4:**
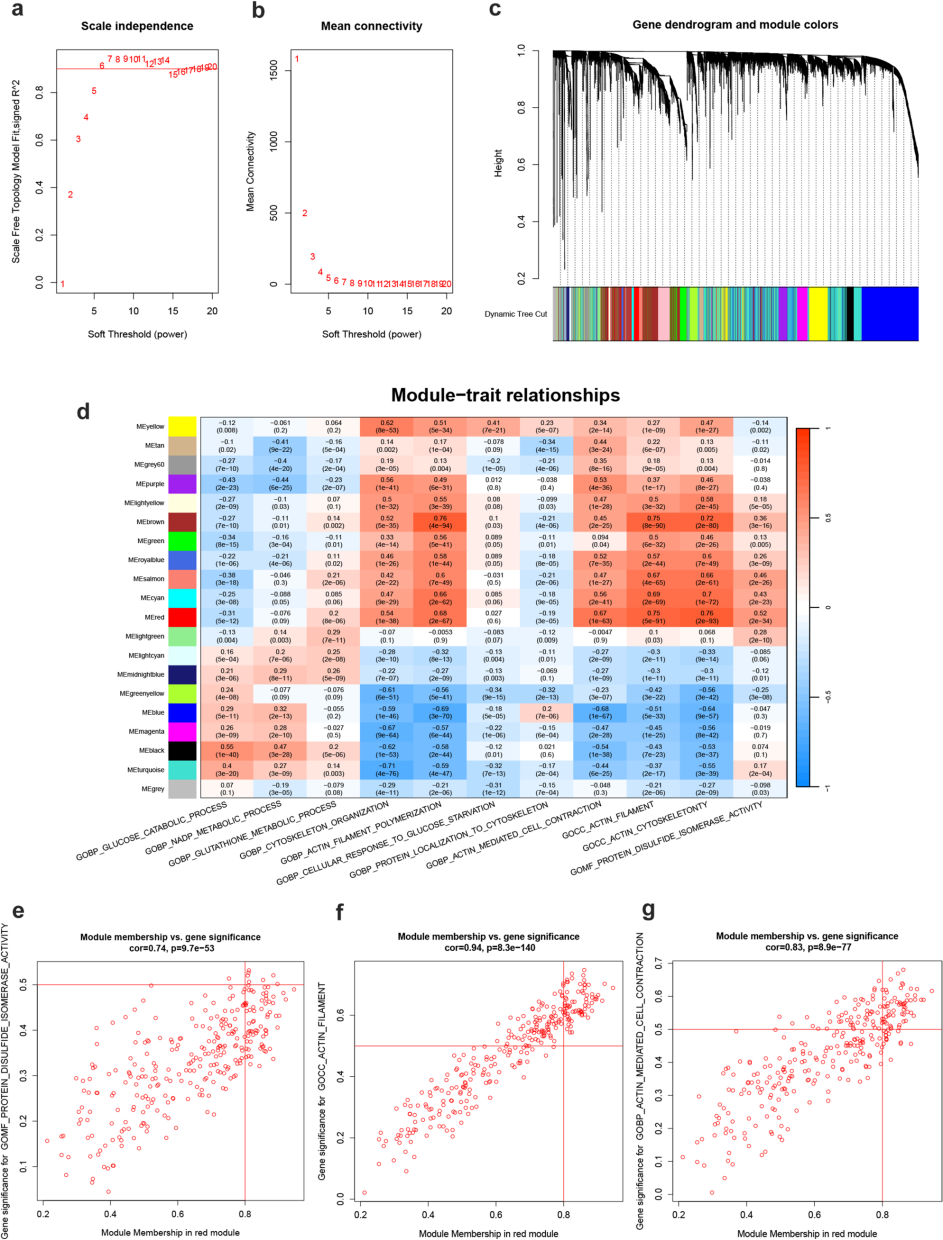
WGCNA analysis of differential genes between cluster 1 and cluster 3. **a**–**b** Analysis of network topology for various soft-thresholding powers, the cut-off R3 value was set to 0.9 and the soft threshold *β* value to 6. **c** Construct co-expression networks based on optimal soft thresholds to divide genes into different modules. **d** Heatmap of correlations between each module and the important pathways in disulfidptosis. **e**–**g** Correlation scatterplot of the red module with the protein disulfide isomerase activity pathway, the actin filament pathway, and the actin-mediated cell contraction pathway

The cut-off R2 value was set to 0.9 and the soft threshold β value to 6 (Fig. 4a–b), under which the network obeyed a power law distribution, which was closer to the real biological network state. Subsequently, a hierarchical clustering analysis based on weighted correlation was performed (Fig. 4c, Sup. 3a–b). A total of 20 modules were identified by WGCNA, with each color representing a different module. Then a heatmap about the module-GSVA pathway trait relationships was plotted according to the Spearman correlation coefficient to assess the association of each module with the important pathways in disulfidptosis (Fig. 4d). Among all the modules, the red module containing 297 genes had a strong correlation with disulfide isomerase activity (cor = 0.74, *p* = 9.7e−53) (Fig. 4e), actin filament (cor = 0.94, *p* = 8.3e−140) (Fig. 4f), actin mediated cell contraction (cor = 0.83, *p* = 8.9e−77) (Fig. 4g), actin cytoskeleton (cor = 0.89, *p* = 1.3e−102), cytoskeleton organization (cor = 0.7, *p* = 4.9e−45), actin filament polymerization (cor = 0.84, *p* = 2.6e−80), and glucose catabolic process (cor = 0.48, *p* = 1.6e−18) (Sup Fig. 3c–h).

### Construction of disulfidptosis prognostic model with multiple machine learning algorithms

**Fig. 5:**
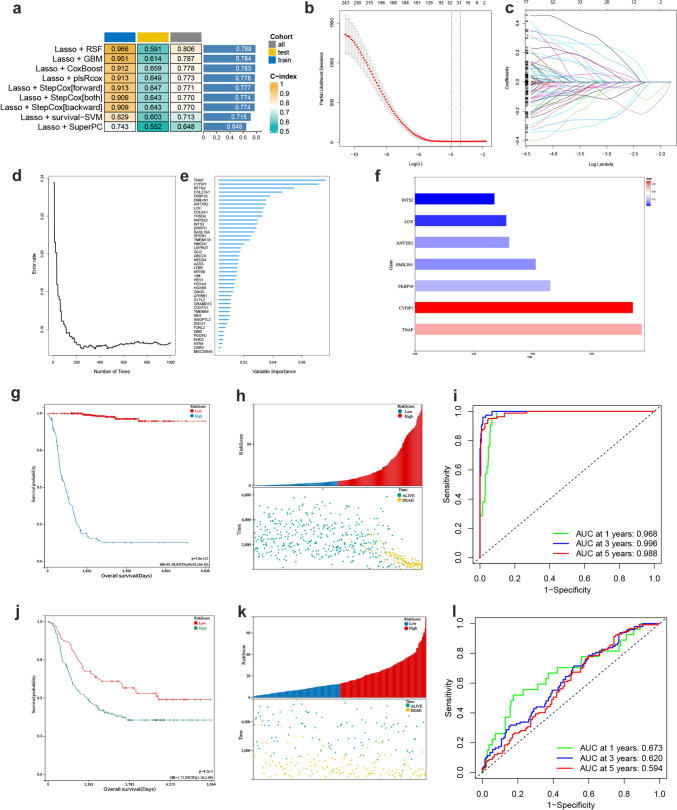
Prognostic models constructed using multiple machine learning algorithms and model validation. **a** Combine LASSO with 9 different machine learning algorithms and compare the C-index. **b** Partial likelihood deviation of LASSO coefficient distribution. The two vertical dashed lines represent *λ*.min and *λ*.1se, respectively, *λ*.min = 0.03335637, *λ*.1se = 0.04064099. **c** Tenfold cross-validation for tuning parameter selection in the LASSO model. **d** Model prediction error rates for different numbers of survival trees. **e** VIMP values for 44 modeled genes. **f** Histogram of genes with the highest VIMP values and their depth in the model. **g** Kaplan–Meier curves for patients in the prognostic high and low scoring groups in the TRAIN dataset. **h** Survival status of patients ranked by risk score in the TRAIN dataset. **i** ROC curves for 1-, 3-, 5-year survival in the TRAIN dataset. **j** Kaplan–Meier curves for patients in the prognostic high and low scoring groups in the TEST dataset. **k** Survival status of patients ranked by risk score in the TEST dataset. **l** ROC curves for 1-, 3-, 5-year survival in the TEST dataset

To identify the signature genes, we utilized the GSE49710 dataset as the TRAIN set, the TARGET dataset as the TEST set, and the merged dataset of both as the ALL set (Sup. 4). Using the LASSO algorithm along with multiple machine learning algorithms in the three cohorts, the C-index of each model was compared. The combination of LASSO and RSF achieved a C-index of 0.788, surpassing other machine learning-based models, indicating superior accuracy and stability (Fig. 5a). In LASSO modeling, 77 prognosis-related genes were identified, gradually reducing as the penalty value λ increased, with the optimal results at λ.min (Fig. 5b–c). Given the impracticality of a large number of genes in clinical detection, further refinement of disulfidptosis prognostic genes while maintaining high accuracy was needed. RSF analysis further reduced the size of independent prognostic genes, achieving a significant decrease in prediction error rate with the increase in survival trees (Fig. 5d). The resultant model comprised 44 genes (Fig. 5e), with TNAP, CYFIP1, FKBP10, EMILIN1, ANTXR2, LOX, and INTS2 as core genes with high variable Importance (VIMP) and depth in the model (Fig. 5f).

### Validation of disulfidptosis prognostic model

NB patients in the 3 datasets were classified into high-risk and low-risk groups based on the optimal cut-off value. Kaplan–Meier curves were plotted (Fig. 5g, j) to evaluate the 1-, 3-, and 5-year survival rates of the two groups, indicating a worse prognosis with higher scores across the TRAIN and TEST cohorts. Patients ranking by score revealed a notable increase in death outcomes with higher scores (Fig. 5h, k). The ROC curves demonstrated the model's accuracy in predicting patient prognosis across different datasets, with satisfactory AUC values (Fig. 5i, l).

### Construction of a nomogram for predicting survival in NB

**Fig. 6:**
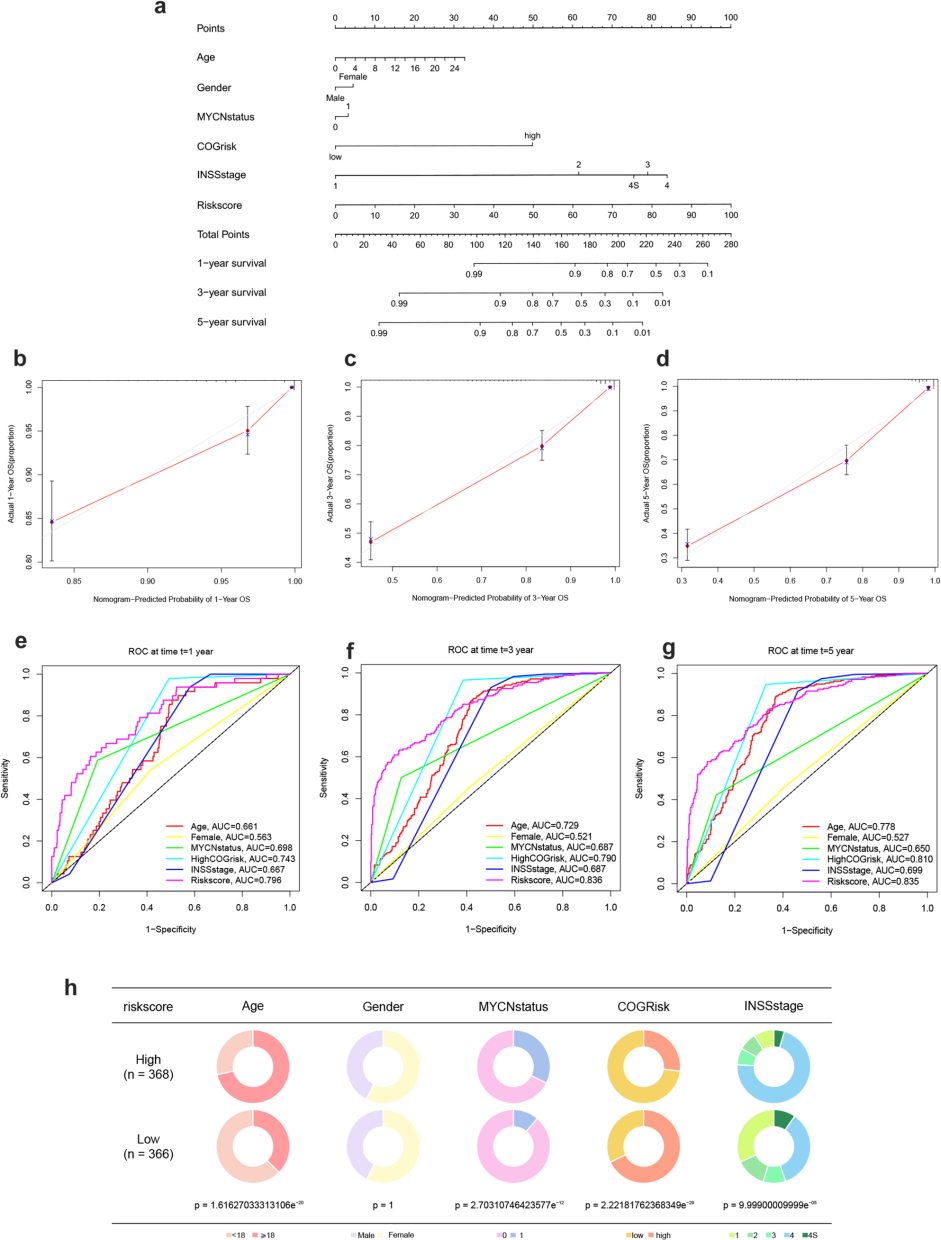
Construction of a nomogram and the evaluation of its effects. **a** The nomogram predicting the risk for NB patients with clinical characteristics. **b**–**d** 1-, 3-, and 5-year calibration curves of the nomogram. **e**–**g** 1-, 3-, and 5-year ROC curves for each characteristic in the nomogram. **h** Pie chart of the percentage of each clinical trait in the high- and low-risk groups

To better predict the survival rates of NB patients, we constructed a nomogram (Fig. 6a) that integrates prognostic models and clinical determinants, including age, gender, N-MYC gene amplification status, COG risk score, and INSS stage. Each item was scored according to the patient's actual condition, and the total score obtained by summing up can be used to predict the survival rate at 1, 3, and 5 years. Subsequently, the 1-, 3-, 5-year predictive accuracy of the constructed nomogram was validated, as shown by the calibration curves (Fig. 6b–d). The red line represents the observed survival rate, while the gray line represents the optimized survival rate, demonstrating a good fit between the observed and optimized values in the model. Additionally, the ROC curves of the nomogram were plotted (Fig. 6e–g), with the area under the curve for age, N-MYC amplification status, COG risk score, INSS stage, and disulfidptosis score being greater than 0.65 at 1, 3, and 5 years. To clearly show the differences in each clinical trait in the high- and low-risk groups, a pie chart of trait percentages was plotted (Fig. 6h).

### Integrated analysis of disulfidptosis modeling genes: functional enrichment, interaction networks, and prognostic assessment

**Fig. 7:**
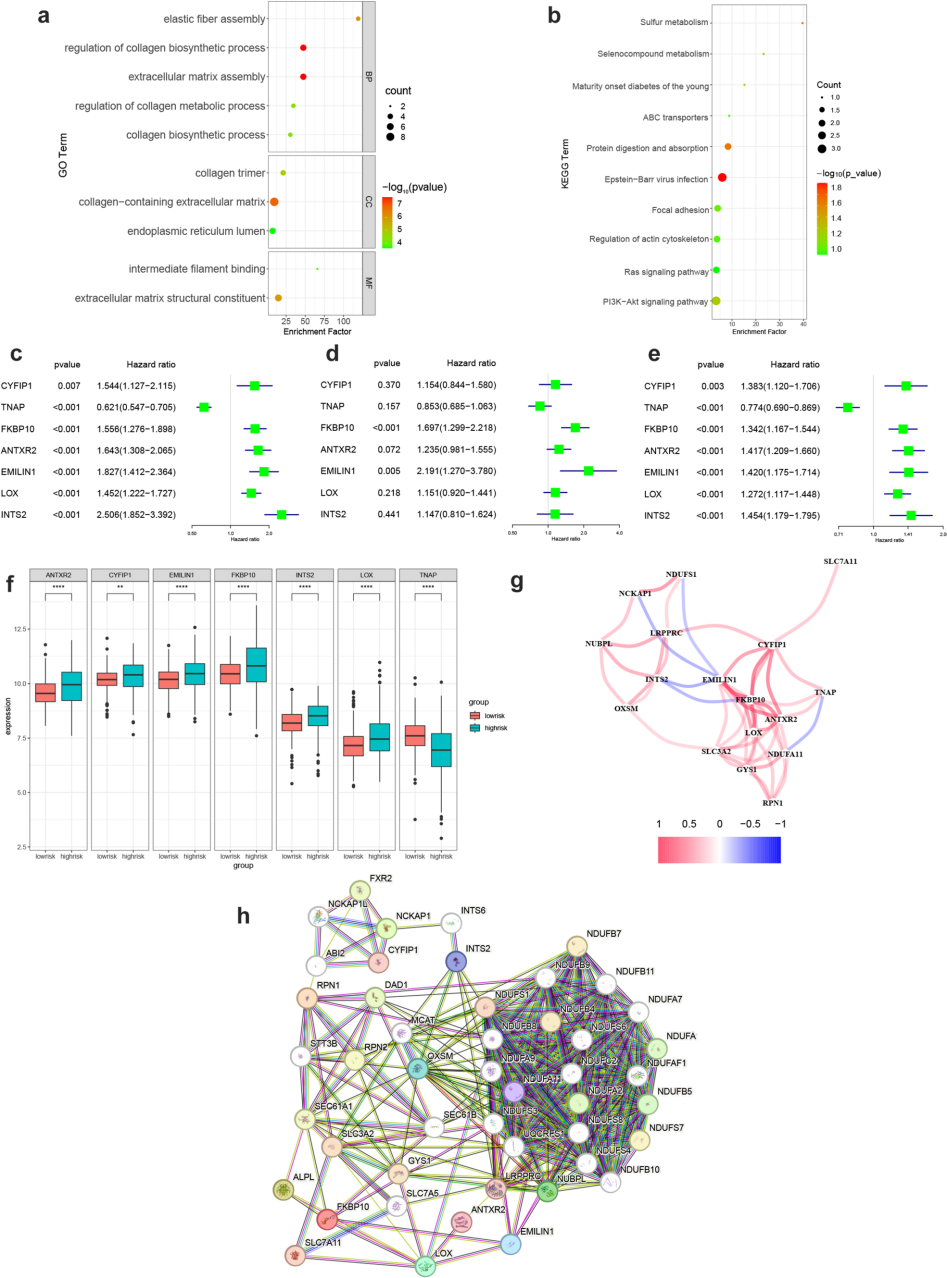
Further analysis of the modeling genes. **a** GO enrichment analysis of 44 disulfidptosis-related modeling genes. **b** KEGG enrichment analysis of 44 disulfidptosis-related modeling genes. **c**–**e** Univariate Cox analysis of 7 core modeling genes in the TRAIN, TEST, and ALL datasets. **f** Box plot of the expression levels of the 7 core modeling genes in the high and low prognostic score groups. **g** Correlation network of core modeling genes with disulfidptosis genes. **h** Protein–protein interaction networks of core modeling genes and disulfidptosis genes

To understand the functional roles and interaction relationships of the modeling genes, GO and KEGG enrichment analysis on 44 disulfidptosis modeling genes was conducted. GO analysis highlighted their involvement in biological processes such as elastic fiber formation, collagen synthesis and metabolism, and extracellular matrix assembly. The enriched components included collagen trimers and extracellular matrix, while the molecular functions were primarily associated with intermediate filament binding and extracellular matrix composition (Fig. 7a). KEGG analysis revealed enrichment in sulfur metabolism, selenium compound metabolism, focal adhesion, regulation of actin cytoskeleton, Ras signaling pathway, and PI3K-AKT signaling pathway (Fig. 7b).

Furthermore, the expression and prognostic impact of 7 core modeling genes were validated within the 3 datasets. ANTXR2, CYFIP1, EMILIN1, FKBP10, INTS2, and LOX exhibited higher expression in the high-risk group (Fig. 7f), serving as risk factors for poor prognosis, with EMILIN1 showing significantly unfavorable prognosis in all 3 datasets, with hazard ratios all > 1.4 (Fig. 7c–e). Conversely, TNAP gene expression was downregulated (Fig. 7f) and acted as a protective factor for prognosis (Fig. 7c–e). The correlations between the 7 core modeling genes and the 10 disulfidptosis genes were examined (Fig. 7g). Red lines indicate positively correlated regulation between the genes, blue lines indicate negatively correlated regulation, and the color shades represent the magnitude of the correlations. CYFIP1 was positively correlated with the genes of SLC7A11, FKBP10, EMILIN1, TNAP, and EMILIN1 and was negatively correlated with the genes of NDUFS1, NCKAP1, and INTS2. To investigate the interaction between the modeling genes and disulfidptosis genes, a protein–protein interaction (PPI) network was constructed using the STRING database (Fig. 7h). The PPI network illustrated clear interactions between the modeling genes and disulfidptosis genes, suggesting that the genes identified in this study may influence disease progression and prognosis in NB by modulating the disulfidptosis pathways.

### Tissue and cellular validation of the mechanism of CYFIP1 and EMILIN1 in tumor phenotype and cytoskeletal alterations

**Fig. 8:**
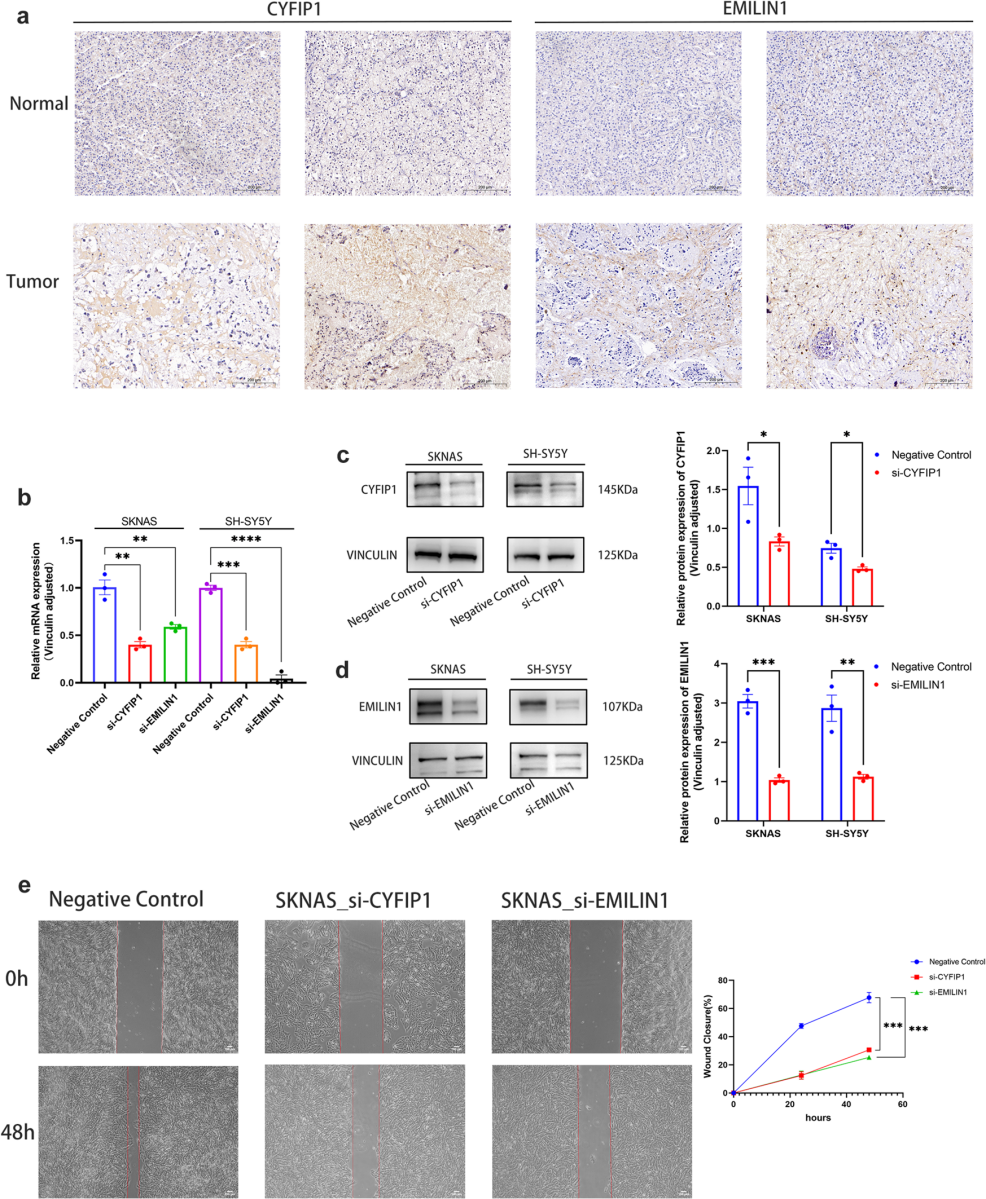

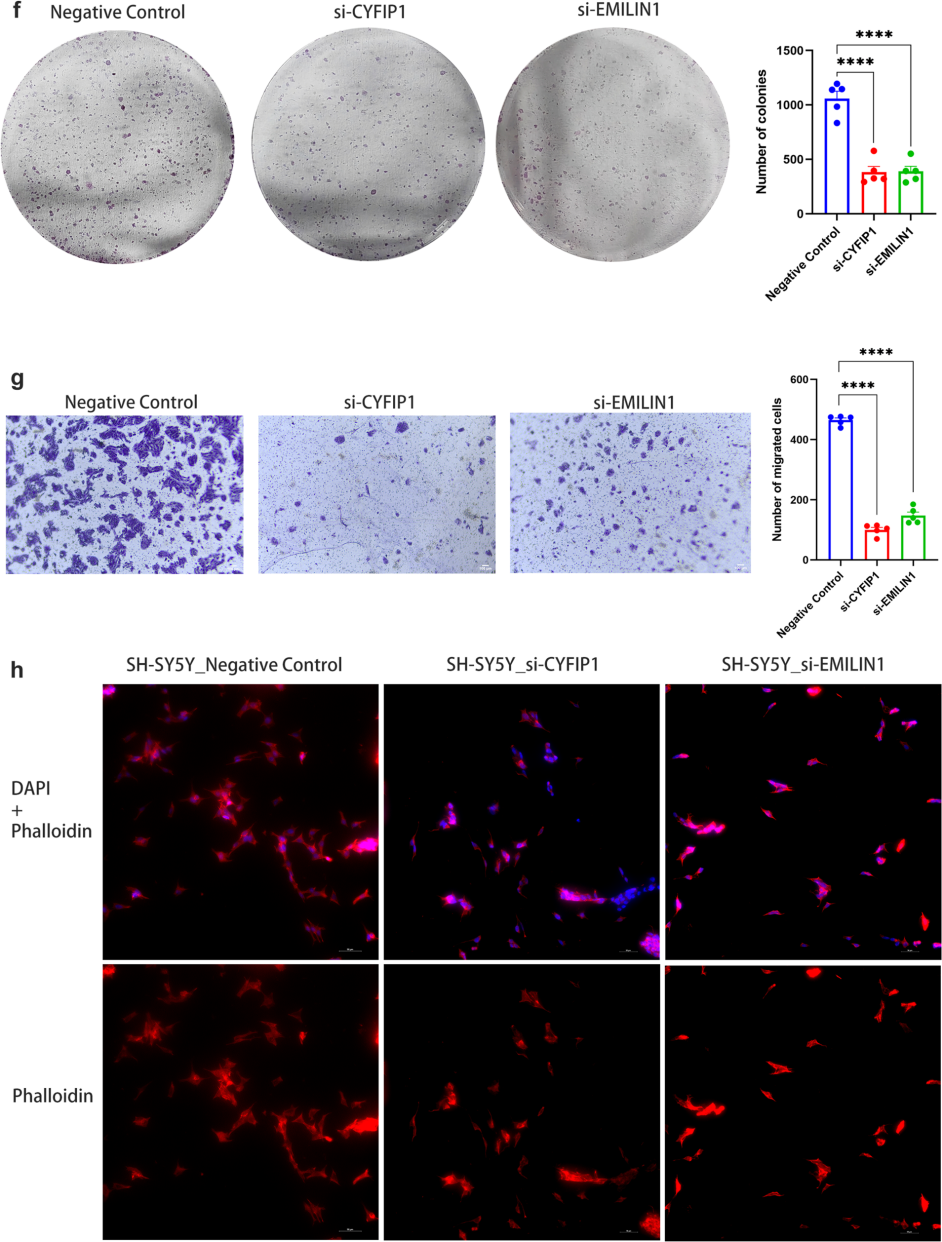
Tissue and cellular experiments to explore the mechanism of CYFIP1 and EMILIN1 genes. **a** Tissue immunohistochemistry revealed elevated expression of CYFIP1 and EMILIN1 in tumor tissue compared to adjacent normal tissue. **b** mRNA expression levels in SKNAS and SH-SY5Y cells in the knockdown and negative control group. **c–d** Protein-level verification of expression differences between negative control and knockdown groups—Western blot bands. **e** Scratch assay confirmed the inhibition of cell migration ability after the knockdown of CYFIP1 and EMILIN1. **f** Clone formation assay confirmed the inhibition of cell colony-forming ability after the knockdown of CYFIP1 and EMILIN1. **g** Transwell assay confirmed the decreased invasive ability of NB cells after the knockdown of CYFIP1 and EMILIN1. **h** Fluorescent staining showed synaptic shortening and disappearance, cytoskeleton distortion, and deformation in NB cells after knockdown of CYFIP1 and EMILIN1. (*p < 0.05, **p < 0.01, ***p < 0.001, ****p < 0.0001 indicate significant differences between the groups shown)

During the modeling process, CYFIP1 exhibited the highest VIMP value, indicating its significant impact on the outcome in the disulfidptosis model and its crucial role in the decision tree for segmentation. Univariate Cox analysis indicated that EMILIN1, not only served as one of the core genes in the construction of the disulfidptosis model but also exhibited high HR across three datasets, indicating an adverse prognostic impact. Consequently, these two genes were chosen for further experimental investigation. Tissue immunohistochemistry revealed an upregulation in the expression of CYFIP1 and EMILIN1 in tumor tissues compared to adjacent normal tissues (Fig. 8a). To further validate the impact of these two genes on NB cell functionality at the cellular level, siRNA transfection was employed to knock down gene expression in SK-N-AS and SH-SY5Y neuroblastoma cells. RT-qPCR (Fig. 8b) and Western blotting (WB) (Fig. 8c–d) demonstrated a significant reduction in the expression levels of both genes in the knockdown group compared to the negative control group, indicating successful inhibition of CYFIP1 and EMILIN1 expression. Subsequent experiments, including scratch assays (Fig. 8e), plate colony formation assays (Fig. 8f), and Transwell assays (Fig. 8g), consistently showed a substantial reduction in the migratory, colony-forming, and invasive abilities of NB cells following CYFIP1 and EMILIN1 knockdown. To investigate the impact of CYFIP1 and EMILIN1 on the cytoskeleton during the process of disulfidptosis, fluorescence staining with DAPI and phalloidin was employed on NB cells from both the negative control and knockdown groups. The outcomes revealed a noticeable reduction and disappearance of NB cell synapses, accompanied by deformations and distortions in the cellular cytoskeleton after the knockdown of both genes (Fig. 8h).

## Discussion

The observed challenges and complexities associated with NB underscore the necessity for a thorough understanding at the molecular level to identify variations that can inform targeted, individualized therapies for high-risk NB patients, ultimately improving prognosis in affected children (Johnsen et al. 2018; Lundberg et al. 2022; Maris 2010). With advancement in high-throughput omics technologies, exploring distinctive molecular expression patterns and seeking out new precision targets stand out as pivotal directions in NB treatment (Jiang et al. 2022).

The pivotal role of RCD in cancer development, governed by diverse factors and signaling pathways, has prompted the exploration of various RCD modes, including apoptosis, necroptosis, ferroptosis, and cuproptosis (Peng et al. 2022; Tong et al. 2022). Among these, disulfidptosis, a novel form of RCD discovered in 2023 (Liu et al. 2023a, b), has drawn attention for its occurrence in cells expressing elevated SLC7A11 levels during glucose starvation. The subsequent accumulation of disulfides, stemming from heightened cystine uptake facilitated by SLC7A11, leads to the formation of disulfide bonds between cytoskeletal proteins, particularly those in the actin cytoskeleton and F-actin (Machesky 2023). This process triggers destabilization, inducing morphological and structural changes in cells. Importantly, disulfidptosis is unaffected by known cell death inhibitors, yet can be induced using glucose transport inhibitors, providing potential solutions to overcome the problem of drug resistance (Zheng et al. 2023a, b). Future studies could focus on disulfidptosis-related signaling pathways such as WAVE protein complex and Rac (WRC) to explore new cancer therapeutic approaches (Zheng et al. 2023a, b).

A growing body of literature has highlighted the significant role of disulfidptosis in various tumors, showcasing its potential as a prognostic marker and therapeutic target. Huang et al. (2023) constructed disulfidptosis-related genetic markers in lung adenocarcinoma and analyzed their impact on the disease and its relationship with the tumor microenvironment. Wang et al. (2023) integrated disulfidptosis with immune infiltration in hepatocellular carcinoma patients, offering new targets and directions for the prognosis and treatment of hepatocellular carcinoma. In addition, the interaction between disulfidptosis, immunity, and metabolism in cancer provides new insights into tumor biology. Zhu et al. (2023) discovered that disulfidptosis significantly affects the activation and infiltration of immune in the tumor microenvironment, suggesting its potential in enhancing immunotherapy for disulfidptosis-sensitive tumors. This also prompts further study into disulfidptosis's role in tumor immune evasion, potentially aiding new immunotherapeutic strategies. Metabolically, disulfidptosis relates to glycolipid metabolism, sulfide metabolism, redox balance, and cellular structures stability. Recent studies have indicated a close relationship between disulfidptosis and metabolic processes, indicating new directions for metabolic NB treatment (Liu et al. 2023a, b; Zheng et al. 2023a, b; Zhang et al. 2023), though detailed molecular understanding and clinical application require more research.

The intricate process of disulfidptosis engages various genes, including GYS1, OXSM, LRPPRC, NDUFA11, NUBPL, RPN1, SLC3A2, SLC7A11, and NCKAP1 (Liu et al. 2023a, b). Specifically, SLC7A11 encodes a cysteine transporter protein, and NCKAP1 contributes to the assembly of the WRC, fostering actin polymerization and the formation of lamellipodia. Focusing on the aforementioned genes, our analysis revealed significant expression differences among NB patients, markedly impacting the prognosis of NB. Based on their expression profiles, GMM clustering identified distinct subtypes, with cluster 3 exhibiting the worst prognosis, marked by tumor progression and frequent N-MYC gene amplification. Differential gene expression and GSVA between clusters revealed significant variations in biological processes such as the construction, transport, and connection of ACTIN cytoskeleton, intracellular glucose homeostasis, cellular responses to glucose deprivation, glucose catabolism, and metabolism. These processes constitute the core components of disulfidptosis, providing further confirmation of its crucial role in NB.

Later, WGCNA was employed to identify modules of genes most correlated with biological processes such as glucose metabolism, cytoskeleton dynamics, and disulfide isomerase activity. Combining LASSO with various machine learning techniques, our preferred algorithm emerged as LASSO coupled with RSF, and this culminated in the construction of a prognostic model comprising 44 genes associated with disulfidptosis. In this prognostic model, higher scores correspond to poorer prognoses. In existing studies on NB prognostic model construction, most researchers use a single algorithm approach for model construction (Huang et al. 2023; Chen et al. 2023; Zhang et al. 2022), while in our study, we integrated multiple machine learning algorithms to select the most suitable one for our sample, enhancing the model's ability to identify complex biomarkers and disease characteristics. Compared to other commonly used machine learning methods, the model constructed by LASSO + RSF has a higher C-index, which indicates superior accuracy and reliability in the evaluation process. The AUC value of this model remains around 0.8 for different forecasting time horizons, including 1 year, 3 years, and 5 years, suggesting that our model has good identification capabilities and is effective in long-term forecasting. Furthermore, our model not only identifies different sample clusters but also assigns each sample to a specific cluster, providing personalized disease risk assessments and treatment recommendations. This personalized prediction enhances treatment accuracy, potentially reducing ineffective treatments, healthcare costs, and improving overall treatment outcomes. In conjunction with clinical characteristics such as age, gender, N-MYC gene amplification status, COG risk, and INSS stage, a corresponding nomogram was constructed, which was evaluated to have strong discriminatory ability and effective prediction, and can be used for clinical diagnosis and treatment of NB. Functional enrichment analysis of the modeling genes revealed their significant involvement in biological processes such as elastic fiber synthesis, formation of the cellular cytoskeleton, collagen synthesis, as well as in pathways related to sulfur metabolism, selenium metabolism, cytoskeleton regulation, and PI3K-Akt signaling.

To further substantiate the role of disulfidptosis in NB, we selected the most distinctive hub genes, CYFIP1 and EMILIN1, for additional histological and cytological experiments. The results confirmed that both CYFIP1 and EMILIN1 were upregulated in tumor tissues, and the knockdown of these two genes affected various tumor phenotypes in NB cells, including colony formation, migration, and invasion. The CYFIP1 gene encodes a protein that is an essential component of the WRC, which promotes actin polymerization, regulates the production of branched actin filaments (Derivery and Gautreau 2010), and also regulates protein translation of NMDAR and related complex components at neuronal synapses to maintain normal synaptic function, morphology, and plasticity (De Rubeis et al. 2013; Hsiao et al. 2016). CYFIP1 dysregulation has been associated with neuropsychiatric disorders, such as intellectual disability, schizophrenia (SCZ), and autism spectrum disorders (ASD) (Kim et al. 2022), and plays an important role in the invasion and metastasis of tumors, such as breast, prostate, and colon cancers (Teng et al. 2016). On the other hand, the EMILIN1 gene encodes an extracellular matrix glycoprotein that plays a role in elastin fiber biogenesis, acting as part of the anchoring filaments connecting the cells to the extracellular matrix (Adamo et al. 2022), which contributes to the fusion of the elastin fibers and makes them more ordered. It has also been shown to play a role in the maintenance of vascular cell morphology, adhesion of smooth muscle cells to elastin fibers, and transforming growth factor (TGFβ) regulation (Randell and Daneshtalab 2017), and has been implicated in diseases such as neuronal, vascular, and valvular lesions (Louzao-Martinez et al. 2019; Munjal et al. 2017). In this study, we also utilized phalloidin staining to visualize the actin cytoskeleton morphology and confirmed that alterations of these two genes can lead to morphological changes in NB cells, including the shortening and disappearance of synapses, as well as deformation and distortion of the cytoskeleton. Functional cell experiments confirmed that downregulation of gene expression could impact cell proliferation, migration, and colony formation. This provides additional evidence that abnormal expression of genes associated with disulfidptosis can influence the functional aspects of NB cells, contributing to the development and progression of NB.

This study has certain limitations that should be acknowledged. Firstly, the number of samples and information used for analysis was limited, and the clinical information sourced from databases may have errors. Secondly, due to experimental conditions and time constraints, the experimental design of this study was not in depth enough, lacking the exploration of the mechanism of disulfidptosis, animal experiments, and large-scale tissue specimen validation. Additionally, our understanding of the interactions between disulfidptosis and immune response and tumor metabolism remains limited, and these findings need to be validated by more intensive and systematic experimental studies. In future research, we will focus on these key questions with the aiming of conducting more comprehensive experiments to further explore the specific mechanisms of disulfidptosis in NB.

## Conclusion

This study revealed elucidates distinct expression patterns and clustering of disulfidptosis in NB, unveiling pathway and prognostic variances among subtypes. A disulfidptosis risk score was successfully constructed, with the ability to accurately predict the prognosis of NB patients. Additionally, key genes (CYFIP1, EMILIN1) associated with disulfidptosis in NB were identified, exhibiting functional roles in cell proliferation, migration, and invasion, leading to notable alterations in the cellular cytoskeleton. Novel therapeutic targets and a refined scoring methods for precision treatment strategies in NB prognosis and management are offered by these findings.

## Supplementary Information

Below is the link to the electronic supplementary material.

Supplementary file1 (DOCX 20 KB)

Supplementary file2 (DOCX 4846 KB)

## Data Availability

The datasets generated during the current study are available from GEO (https://www.ncbi.nlm.nih.gov/geo/) and TARGET (https://www.cancer.gov/ccg/). More detailed data are available from the corresponding authors upon reasonable request.
