# Supplementary material for: Integrated machine learning-driven disulfidptosis profiling: CYFIP1 and EMILIN1 as therapeutic nodes in neuroblastoma
Source: J Cancer Res Clin Oncol. 2024 Mar 1;150(3):109. doi: 10.1007/s00432-024-05630-8 (PMC10907485; doi:10.1007/s00432-024-05630-8)
Supplement: Supplementary file 1 — Supplementary file1 (DOCX 20 KB) [file 432_2024_5630_MOESM1_ESM.docx]

**297 gene in the Red module:**

ZFP36L1

ZBTB47

YAP1

WWTR1

WWC3

WWC2

WISP1

WIPI1

WFDC1

VIM

VCL

VCAN

UACA

TTMB

TRPM4

TRIOBP

TRAM2

TPM1

TNS1

TNN

TNFRSF1A

TNFRSF12A

TNAP

TMEM54

TMEM51

TMEM47

TMEM26

TMEM16A

TMEM133

TM4SF1

TIMP3

THSD4

TGFBI

TGFB2

TFPI2

TENC1

TCF7L1

TBC1D8B

TAGLN

SYTL2

SYNJ2

SVIL

SUSD2

STAB1

SPON2

SPON1

SPARC

SOX13

SNX21

SLC6A1

SDC2

SASH1

S100A16

RRBP1

RRAS

RNF128

RHOC

RHBDF1

RGL3

RFTN2

RCAN2

RASL10A

RAB34

RAB31

PZP

PVRL2

PTRF

PTPRB

PTPN14

PTHR1

PRKCDBP

PPIC

PPAP2B

PPAP2A

PON2

PLXDC2

PLXDC1

PLS3

PLK2

PLEKHH2

PLAT

PLAC9

PHLDB3

PHLDB2

PDGFRL

PDGFRB

PDGFD

PDE1A

PARD3B

PAPSS2

PAIP2B

OSR2

OLFML2B

OLFML2A

ODF3L1

OAF

NTN4

NR2F2

NPNT

NOTCH2

NID2

NID1

NEXN

NES

NEDD4

MYO10

MYLK

MYL9

MYH9

MYH11

MXRA8

MUSTN1

MUC20

MT2A

MT1X

MT1H

MT1F

MT1A

MRCL3

MOSC2

MN1

MICALL2

MICAL2

MGC4294

MGC39545

MAP3K6

LYPD6

LTBR

LRRC32

LRP10

LPP

LOX

LOH3CR2A

LOC651721

LOC645954

LOC54492

LOC541471

LOC389033

LOC349160

LOC340508

LOC286044

LOC285047

LOC253264

LMNA

LMCD1

LHFP

LGALS1

LEPROT

LEPREL1

LEPRE1

LCAT

LATS2

LAMB2

LAMA4

KRT80

KIRREL

KIAA1217

KCNN1

KCNJ11

KCNE4

KCNB2

KAZALD1

JUB

JAG1

ITGB5

ITGB1

ITGAV

ITGA7

INTS2

INPPL1

IL1RAP

IGFBP7

IGFBP5

IGFBP4

IGF2

HTRA1

HSPB7

HSPB3

HOXB9

HOXA5

HOXA4

HNT

HMCN1

HEYL

HES1

HEPH

GSN

GRAMD1C

GPR116

GNG5

GNG12

GLI3

GLI2

GEM

GAS2L1

FOXF2

FNDC1

FLJ42393

FLJ23556

FLJ10357

FKBP10

FILIP1L

FER1L3

FCGRT

FBXO32

FBN1

FBLIM1

FAM38A

F2RL2

EVC

ERRFI1

EPS8

EPHB4

ENPP1

ENG

EMP1

EMILIN1

EHD2

ECM2

ECM1

DUSP6

DSP

DPF1

DOCK6

DOCK1

DMD

DKFZP564O0823

DCBLD1

DACT1

DAB2

DAAM2

CYFIP1

CYB5R2

CTHRC1

CTGF

CSDA

COX7A1

COL6A3

COL6A2

COL6A1

COL5A3

COL4A2

COL4A1

COL3A1

COL27A1

COL21A1

COL1A2

COL1A1

COL18A1

COL16A1

COL15A1

COL12A1

COBLL1

CNN1

CMTM3

CLIC4

CEBPD

CDH13

CDH11

CD163

CD109

CCDC102B

CASQ2

CA2

C9orf47

C6orf52

C6orf145

C3orf57

C1orf198

C1orf136

C18orf51

C14orf162

C10orf72

C10orf47

BDKRB2

AXL

ATP8B1

ATP10A

ASPN

AOC3

ANXA5

ANXA1

ANTXR2

ANTXR1

ANKRD25

ANGPTL2

ALDH1A3

AHNAK

AGT

AEBP1

ADRA1B

ADAMTS12

ACACB

ABCG2

ABCC9

AASS

A2M

**44 modeling genes:**

VIM 0.0139832909516969

TNAP 0.0753292248083154

TMEM54 0.0111352986137314

TMEM133 0.0232038318409883

THSD4 0.0309299422349041

SYTL2 0.0104465159271456

SPON1 0.0253210450518433

SNX21 0.00883084316217123

RFTN2 0.0538117173568125

RASL10A 0.0260614632800866

PDGFD 0.00650683059093369

PAPSS2 0.0287291682175367

OSR2 0.00440681332979212

NTN4 0.00576168275935407

NES 0.00881526332806053

NEDD4 0.0169263135838333

MGC39545 0.00315194151024098

LTBR 0.0137626712795855

LOX 0.0343442458839341

LEPROT 0.0175576179736674

KRT80 0.0153401273047798

INTS2 0.028446524255629

HOXB9 0.0129306944289085

HOXA4 0.0148487139819935

HMCN1 0.0188795584149914

HES1 0.0131424763968374

GRAMD1C 0.00998139676228615

GNG5 0.0117113600639083

GLI2 0.0156833220823299

FKBP10 0.0377322583061563

F2RL2 0.00853448862240137

ERRFI1 0.028960766365479

EMILIN1 0.0349337242863128

EHD2 0.00536709846256715

DMD 0.00607896819912129

CYFIP1 0.0722558714097722

COX7A1 0.0103744942608734

COL6A1 0.0328083805090547

COL27A1 0.0448391738012139

ATP8B1 0.0112216959680622

ANTXR2 0.0353544466192322

ANGPTL2 0.00889865451411499

ABCC9 0.017416051448493

AASS 0.0148641747842681
