# Supplementary material for: Integrated machine learning-driven disulfidptosis profiling: CYFIP1 and EMILIN1 as therapeutic nodes in neuroblastoma
Source: J Cancer Res Clin Oncol. 2024 Mar 1;150(3):109. doi: 10.1007/s00432-024-05630-8 (PMC10907485; doi:10.1007/s00432-024-05630-8)
Supplement: Supplementary file 2 — Supplementary file2 (DOCX 4846 KB) [file 432_2024_5630_MOESM2_ESM.docx]

**Construction of Disulfidptosis-related molecular subtypes and prognostic signature in neuroblastoma with multiple machine learning**

Zhang Mengzhen^&^, Hou Xinwei^&^, Tan Zeheng^&^, Yang Yang, Li Nan, Fan Kaisi, Ding Xiaoting, Yang Huirong, Yang Liucheng*, Wu Kai*


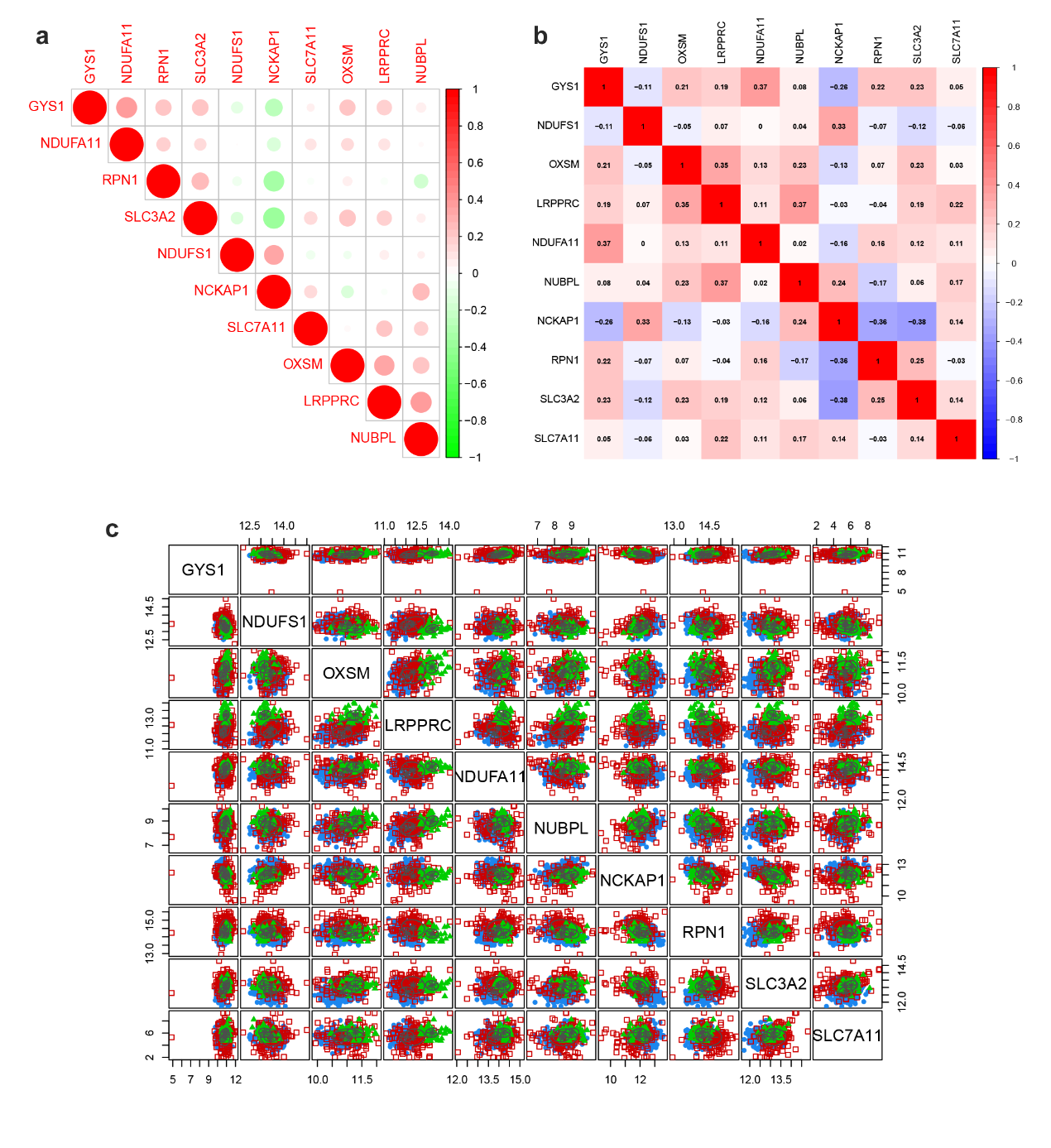


**Fig. S1** Expression of disulfidptosis genes in NB samples and in clusters. **(a)-(b)** Correlation heatmaps of interactions between disulfidptosis genes in the GSE49710 dataset. **(c)** Expression distribution of disulfidptosis genes in 3 molecular subtype samples.

**
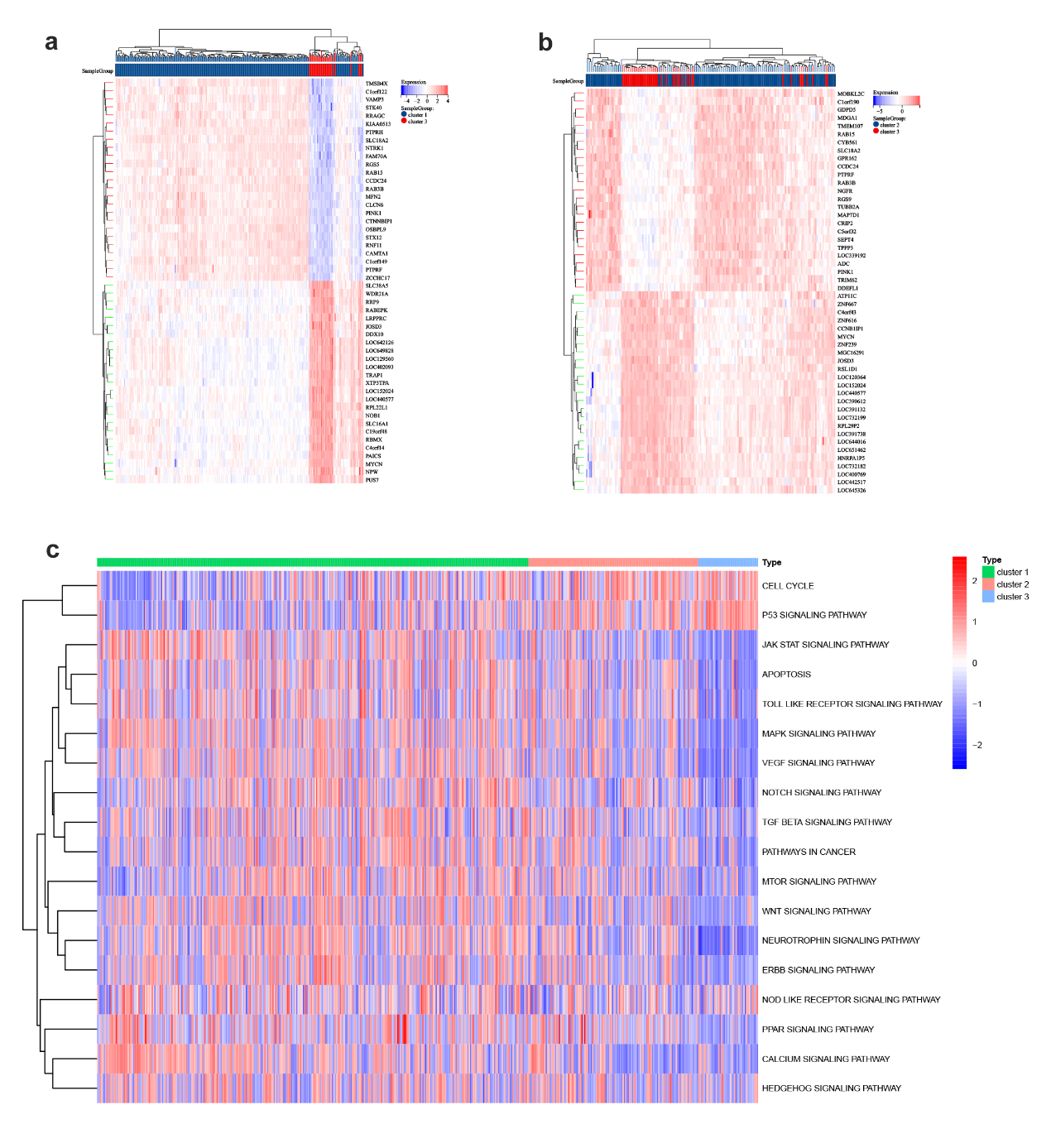
**

**Fig.S2** Differential gene analysis and tumor-associated GSVA pathway analysis between cluster. **(a)-(b)** Top50 genes with up- or down-regulated expression between clusters. **(c)** Heatmap of Classical cancer-related signaling pathways expression in 2 molecular subtype samples.


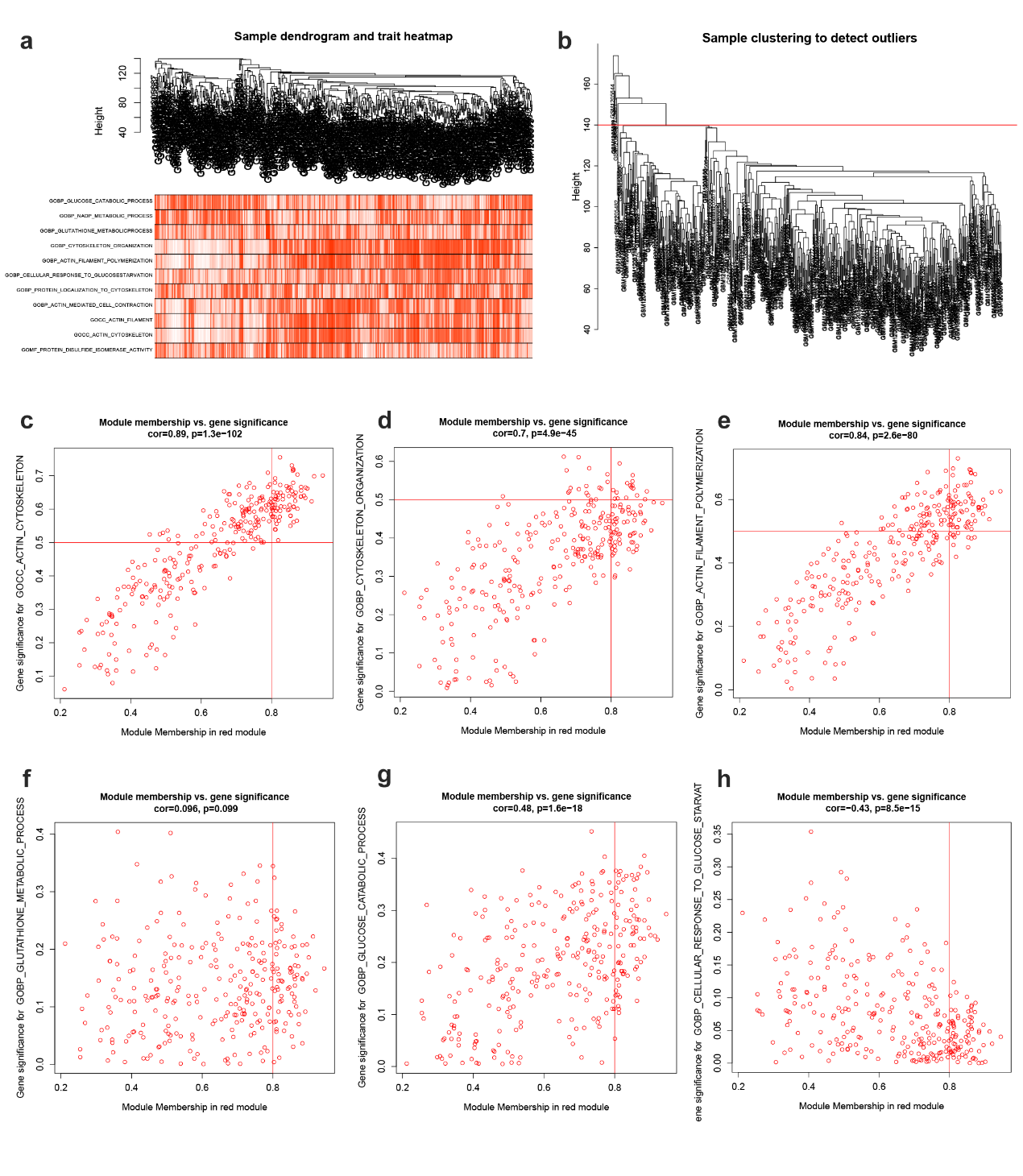


**Fig.S3** Correlation of WGCNA gene modules with the disulfidptosis pathway. **(a)** sample dendrogram and trait heatmap in co-expression networks. **(b)** Sample clustering in co-expression networks. **(c)** Correlation scatterplot of the red module with the actin cytoskeleton pathway. **(d)** Correlation scatterplot of the red module with the cytoskeleton organization pathway. **(e)** Correlation scatterplot of the red module with the actin filament polymerization pathway. **(f)** Correlation scatterplot of the red module with the glutathione metabolic process pathway. **(g)** Correlation scatterplot of the red module with the glucose catabolic process pathway. **(h)** Correlation scatterplot of the red module with the cellular response to glucose starvation pathway.


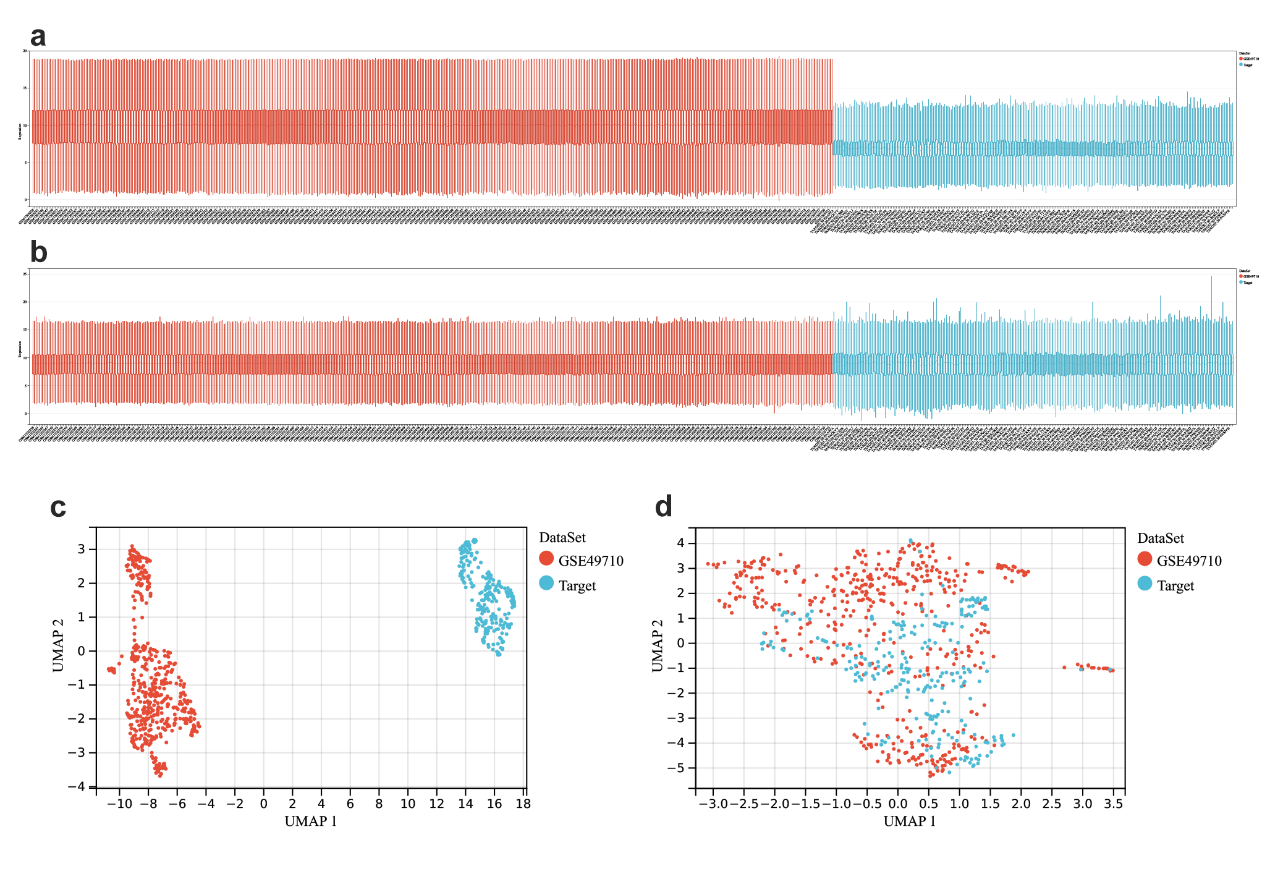


**Fig.S4** The GSE49710 and TARGET datasets were merged and the ALL dataset was obtained by removing the batch effect. **(a)-(b)** Boxplots of data distribution for GSE49710 and TARGET dataset before and after removing batch effects. **(c)-(d)** UMAP plots of data distribution of GSE49710 and TARGET dataset before and after removing batch effects.
